# Supplementary material for: Factorial structure, validity, and gender invariance of the UCLA-R loneliness scale in ecuadorian adolescents
Source: PLoS One. 2025 Aug 4;20(8):e0328163. doi: 10.1371/journal.pone.0328163 (PMC12321113; doi:10.1371/journal.pone.0328163)
Supplement: Attachment — Submitted filename: Response to reviewers.docx [file pone.0328163.s001.docx]

**RESPONSE LETTER**

**Response to the Editor’s Comments**

1. **Comment:** Ensure your manuscript complies with PLOS ONE’s style requirements, including file naming conventions.

**Response:** The manuscript has been reviewed and adjusted according to PLOS ONE guidelines.

2. **Comment:** Ensure the title matches that of the manuscript and the cover letter.

**Response:** The title in the cover letter has been adjusted accordingly.

3. **Comment:** We noted that the grant information provided in the "Funding Information" and "Financial Disclosure" sections does not match.

**Response:** The issue regarding the financial grant has been clarified.

4. **Comment:** The repository name is missing in your Data Availability Statement.

**Response:** The name of the data repository has been added.

5. **Comment:** In the submission form, you indicated that the data would be made available upon acceptance.

**Response:** The anonymized database has been made openly accessible for general use.

6. **Comment:** We observed that the supplementary file "Complete Ucla" contains identifiable data.

**Response:** The database has been adjusted and anonymized.

7. **Comment:** Include the titles of your supplementary files at the end of your manuscript and update in-text citations to match.

**Response:** No supplementary files are included.

**Response to Reviewer 1**

1. **Comment (Title):** In my humble opinion, "Gender invariance" would be preferable to "sex differences." If agreeable, please revise the terminology throughout the manuscript.

**Response:** The suggestion has been considered, and the title and relevant sections of the manuscript have been updated as requested.

2. **Comment (Abstract):** Typographical error: "objective" instead of "objective."

**Response:** The typographical error has been corrected.

3. **Comment (Abstract):** The objective should provide more details about the different analyses conducted to validate the scale.

**Response:** The objective has been revised as suggested.

4. **Comment (Methods):** The methods section is too brief and lacks sufficient detail. Enrich it with necessary information (e.g., mean age and standard deviation, sociodemographic variables, sample proportions for EFA and CFA).

**Response:** The section has been expanded as requested.

5. **Comment (Results):** Provide the findings from the EFA.

**Response:** The EFA findings have been included.

6. **Comment (Introduction):** Add a paragraph on the need for cross-cultural validation. Why is it important to use a scale validated in the local language?

**Response:** A paragraph addressing this has been added.

7. **Comment (Procedure):** How did you attempt to limit respondent bias? Did you use a guideline for online surveys (e.g., CHERRIES, Eysenbach, 2012)? If not, explain your procedure.

**Response:** The procedure to limit bias in online surveys has been added.

8. **Comment (Results):** I suggest removing Table 2, as the necessary information is already included in the text. Otherwise, it would be redundant.

**Response:** Table 2 has been removed, and the numbering of subsequent tables has been adjusted.

9. **Comment (Results):** Provide findings related to the normality of the distribution (Skewness and Kurtosis) evaluated in Table 3 and add them to the table title.

**Response:** A paragraph addressing skewness, kurtosis, mean, and standard deviation has been added.

10. **Comment (Discussion):** I recommend adding paragraphs on the practical and theoretical implications of the study.

**Response:** Paragraphs on practical and theoretical implications have been included.

11. **Comment (Limitations):** Additional limitations should be added, such as bias in online surveys or the limited sample size. Explain why convergent or divergent validity was not assessed.

**Response:** Additional limitations and explanations have been provided.

12. **Comment (Conclusion):** Please rewrite this section, as it currently reiterates results. The conclusion should focus on answering the primary objective of the study, so reporting GFI results is unnecessary.

**Response:** The conclusion has been revised accordingly.

**Response to Reviewer 2**

1. **Comment (Line 20):** The sentence suggests that the bifactor model does not vary between genders in terms of measurement invariance. It could be phrased more clearly.

**Response:** The text has been adjusted for clarity.

2. **Comment (Lines 102–103):** Could you briefly explain this methodology? Is it similar to simple random sampling?

**Response:** The methodology has been clarified.

3. **Comment (Lines 216–217):** Provide explanations for why this item did not load onto any factor.

**Response:** An explanation has been added.

4. **Comment (Table 5):** Calculating alpha reliability for each dimension and stratified alpha coefficients for the entire scale would provide a more precise lower bound of alpha reliability for multidimensional scales.

**Response:** The requested values have been included.

5. **Comment (Line 274):** You could reference why it is more accurate to observe differences between these indices, as the chi-square statistic is affected by sample size.

**Response:** A reference and explanation have been added.

6. **Comment (Line 338):** You mentioned removing an item because it did not sufficiently apply to any factor. It would be helpful to explain why this item did not work with adolescents. Has this issue been observed in other adaptation studies?

**Response:** A paragraph addressing this has been added to the discussion.
